# Carbon Monoxide: A Pleiotropic Redox Regulator of Life and Death

**DOI:** 10.3390/antiox13091121

**Published:** 2024-09-16

**Authors:** Andrey Y. Abramov, Isabella Myers, Plamena R. Angelova

**Affiliations:** UCL Queen Square Institute of Neurology, Department of Clinical and Movement Neurosciences, Queen Square, London WC1N3BG, UK; a.abramov@ucl.ac.uk (A.Y.A.); isabella.myers@outlook.com (I.M.)

**Keywords:** carbon monoxide poisoning, CO toxicity, reactive oxygen species, oxidative stress, neuron, cell death

## Abstract

Despite recent technological progress, carbon monoxide poisoning is still one of the leading causes of domestic and industrial morbidity and mortality. The brain is particularly vulnerable to CO toxicity, and thus the majority of survivors develop delayed movement and cognitive complications. CO binds to haemoglobin in erythrocytes, preventing oxygen delivery to tissues, and additionally inhibits mitochondrial respiration. This renders the effect of CO to be closely related to hypoxia reperfusion injury. Oxygen deprivation, as well as CO poisoning and re-oxygenation, are shown to be able to activate the production of reactive oxygen species and to induce oxidative stress. Here, we review the role of reactive oxygen species production and oxidative stress in the mechanism of neuronal cell death induced by carbon monoxide and re-oxygenation. We discuss possible protective mechanisms used by brain cells with a specific focus on the inhibition of CO-induced ROS production and oxidative stress.

## 1. Introduction

Carbon monoxide (CO) is an odourless, colourless, tasteless, poisonous gas produced when fuels containing carbon burn without sufficient oxygen. It is often associated with fire incidents as a component of smoke along with other products of combustion, causing respiratory and cardiovascular injury, and in sufficient concentrations, death. CO is the component of smoke that ultimately causes death in fire incidents, due to its presence in high volumes. However, fires are not the only situations where CO is produced; malfunctioning or inappropriately used household appliances that are fuelled by, for example, gas, coal, wood, oil, or diesel, will also produce CO and can leak into indoor spaces from cracked, blocked, or broken chimneys and flues. These scenarios can result in the presence of this invisible killer going unnoticed, resulting in the unintentional poisoning and, in some cases, death of occupants. Case studies have highlighted other sources of CO leading to indoor exposure to this gas when shisha pipes, BBQs, and generators are inappropriately used indoors or poorly positioned.

CO alarms are available to help detect the presence of this gas, sounding at levels that are considered imminently dangerous to life. Developments in the scientific literature have shown that chronic exposure to levels that are not lethal and that produce mild, non-specific symptoms can also cause illness and impact on health. Diagnosis of CO exposure and poisoning is considered challenging due to the non-specific symptoms it produces, yet it is still one of the leading causes of unintentional toxic mortality and morbidity [1]. One serious problem associated with CO poisoning is that a high percentage of survivors—up to 70%—develop neurological and psychiatric sequalae, such as cognitive decline and movement abnormality, and are often misdiagnosed with dementia.

The basic mechanism of CO toxicity has been known for decades; it binds with haemoglobin (CO interacts with Fe^2+^ from the heme of haemoglobin and myoglobin) in erythrocytes, forming carboxyhaemoglobin, thus preventing the binding of oxygen to erythrocytes and the release of any already bound oxygen, which leads to oxygen deprivation in the tissues [2]. In addition to hypoxic/anoxic conditions, carbon monoxide binds to and inhibits cytochrome C, which blocks the mitochondrial electron transport chain [3]. Consequently, oxygen consumption in mitochondria is blocked, which can be seen as a form of chemical hypoxia. All of these factors drive strong associations of CO poisoning to hypoxic/anoxic types of injuries. It should also be added that CO can bind and change the function of the cytochrome P450, heme-based O_2_ and NO sensors, and other proteins that may have an effect on redox balance [4,5,6].

Therefore, the first step in the treatment of CO poisoning is the re-introduction of oxygen to displace the CO in erythrocytes. In many countries, the provision of hyperbaric oxygen is considered the most effective means of treatment due, in part, to its ability to accelerate the excretion of CO; it reduces the half-life of CO from 4.5 to 5.0 h when breathing air to about 20 min [7]. Toxicokinetics of COHb (carboxyhaemoglobin) are still disputable and can be estimated by the COHb half-life, which differs depending on the level of surrounding oxygen [8]. However, the process of re-oxygenation, which is known to be quite destructive immediately after hypoxia/anoxia, has been found to be even more damaging as a measure of recovery for patients with CO poisoning, although similar studies using hyperbaric oxygen for the treatment of CO poisoning are lacking. Despite the apparent simplicity of the mechanisms of CO poisoning, the molecular mechanisms of cellular, and particularly neuronal death associated with poisoning and reperfusion injury, are not fully understood, although a growing number of publications indicate oxidative damage as being the major trigger for neuronal loss [9].

Whilst CO is a product of the inefficient burning of fuels containing carbon and high-temperature-derived industrial vapours, it is important to acknowledge that CO is continuously produced endogenously in cells by the enzyme haem oxygenase; the enzyme responsible for haemoglobin homeostasis [10]. Heme oxygenase isoforms, described as HO-1 and HO-2 (also known as HSP-32), catalyse the oxidation of heme, the prosthetic group of hemoproteins, using the energy of NADPH into biliverdin with divalent iron (Fe^2+^) and carbon monoxide. Biliverdin is then reduced by biliverdin reductase into bilirubin, which is the final product of heme metabolism [11,12]. However, the byproduct of this reaction**,** CO, is actively used as a gasotransmitter and is proven to exert an important role in the regulation of many physiological processes [10]. As a gasotransmitter, it actively interacts with its “chemical siblings”, nitric oxide and hydrogen sulphide (and other forms of reactive oxygen species), in endothelial cells [13,14,15]. Considering the important role of HO-1 in the maintenance of heme homeostasis and in the regulation of CO levels, the expression of HO-1 in the brain is present at surprisingly low levels in glia and neurons of the dentate gyrus of the hippocampus, the hypothalamus, and some brainstem areas. HO-2 is the most abundant in the brain, which possibly compensates for the low level of HO-1 in this organ [11,12]. Endogenously produced CO by HO-1 or HO-2 acts as a neuroprotectant [16].

Reactive oxygen species are constantly produced enzymatically and non-enzymatically in cells and play the role of signal transmitters in redox signalling processes [17,18]. Redox balance is maintained by an efficient antioxidant system that protects biological molecules against oxidative stress. Redox signalling is a system very sensitive to changes in cellular function. Thus, energy deprivation and changes in nucleotide balance induce the activation of xanthine oxidase, which produces hydrogen peroxide and changes in glucose metabolism or oxygen levels. This, in turn, leads to changes in ROS production from the mitochondrial electron transport chain (ETC) [19], the release of neuromodulator monoamines such as adrenaline or dopamine, and the activation of H_2_O_2_ production in monoaminoxidases (MAOs) [20]. It should be noted that the enzymes of the NADPH oxidase family produce the highest amounts of ROS in physiology and pathology. Combined activation of these enzymes, leading to a pathological decrease in antioxidant levels has been shown to be the trigger of oxidative damage in a number of pathologies, including ischemia/reperfusion injury.

Despite its relatively small size (~2% of body mass), the brain consumes about 20% of total oxygen and 20% of all glucose, making brain tissues the most sensitive tissue to oxygen/glucose deprivation. Neurons, in contrast to any other cell types in the body, are postmitotic cells; therefore, their death is irreversible and leads to the loss of cognitive or movement function. Additionally, the rate of energy metabolism and redox balance, including the levels of the major endogenous antioxidants and the effectivity of the major ROS-producing enzymes, varies in different brain regions, which could also possibly determine the differential vulnerability of neurons from these regions to CO-induced toxicity [21,22].

Here, we review the role of reactive oxygen species production and oxidative stress in the mechanism of brain cell loss in CO poisoning and subsequent ischaemia/reperfusion injury. We discuss the role of various enzymes in oxidative damage, the difference between anoxic and CO neurotoxicity, and why the re-introduction of oxygen is particularly toxic for brain cells. Finally, we discuss a progressive view on potential therapeutic strategies for the treatment of CO poisoning.

## 2. Physiological Role of CO

A wide range of physiological functions of endogenously produced CO include vasomodulation, anti-inflammatory, anti-apoptotic, and bioenergetic (metabolic) functions and a role in cell proliferation [23]. Physiological CO-related processes are mediated by ROS, mainly produced in the mitochondria, where CO stimulates the tricarboxylic acid cycle (TCA)’s efficiency and oxidative phosphorylation (OXPHOS), thus boosting energy production [24]. Additionally, CO suppresses glycolysis and increases the efficiency of the pentosophosphate pathway (PPP) [25,26].

**Figure 1:**
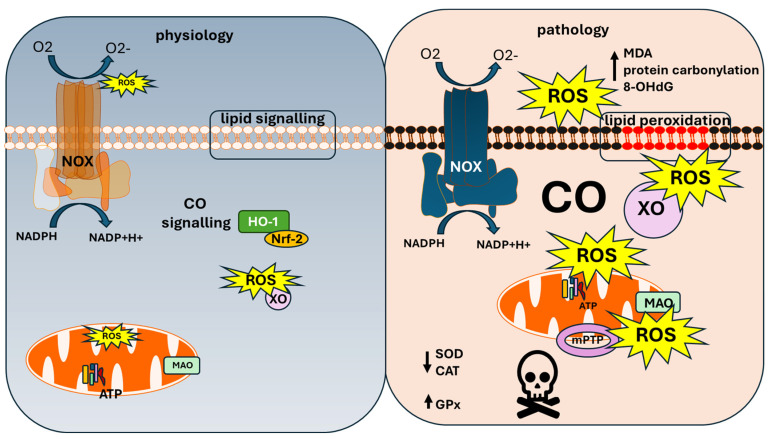
**The role of carbon monoxide in physiology and pathology.** Left: at physiological conditions of the cell, CO is produced in the HO-1 and enables cell detoxification and signalling processes. Right: at pathologically acute high doses of CO-NOX, XO and mitochondria produce ROS with higher rates, which leads to lipid peroxidation and mPTP opening and, ultimately, to cell death. CO—carbon monoxide; ROS—reactive oxygen species; NOX—NADPH oxidases; HO-1—heme oxygenase; Nrf-2—nuclear factor erythroid 2-related factor 2; XO—xanthine oxidase; MAO—monoamine oxidase; NADPH—reduced nicotinamide adenine dinucleotide phosphate, NADP+—oxidised nicotinamide adenine dinucleotide phosphate; ATP—adenosine 3-phosphate; mPTP—mitochondrial permeability transition pore; MDA—malone dialdehyde; 8-OHdG-8-hydroxy-2′-deoxyguanosine; SOD—superoxide dismutase; CAT—catalase; GPx—glutathione peroxidase.

At low, endogenously produced doses, CO plays a cytoprotective role as an inductor of mitophagy, autophagy, mitochondrial biogenesis, and a modulator of calcium channels which prevent excitotoxicity-related neuronal cell death [27]. CO also stimulates guanylate cyclase [28] and takes part in cellular paracrine signalling. These cytoprotective effects are mediated through the activation of the MAPK, PI3K/Akt, and STAT3 pathways [29]. HO-1 is controlled by nuclear factor erythroid 2-related factor 2 (Nrf-2) activation; thus, physiological CO release is directly dependent on the redox state of the cell [30]. Nrf-2 is the main regulator of the expression of proteins of antioxidant and detoxifying systems. Moreover, it controls energy metabolism and production of ROS in the mitochondria and in NADPH oxidase [31,32,33]. Considering the tight interaction between HO-1 (Hsp-32) and Nrf-2, this suggests the regulation of the expression of HO-1 by ROS via oxidative stress, and the regulation of cellular redox homeostasis by HO-1 (see Figure 1).

It is, therefore, not surprising that in the case of acute exposure of cells to high doses of exogenous CO (i.e.**,** from poorly combusting fuel), CO will attack the same pathways and bind with the highest affinity to its physiological targets. The toxicity of high doses of CO is substantiated by the extremely high affinity of CO to multiple targets, mainly carrying transition metals, such as haem-containing proteins, as found in many enzymatic active centres of proteins with porphyrin structures, i.e., cytochrome C oxidase from the ETC, cytochrome P450-dependent monooxygenases (CYPs), NADPH oxidases, etc., which results in the inactivation/overactivation of these enzymes. In addition to haem-containing enzymes, CO binds with a high affinity to other haemoproteins such as transcript factors, ion channels, transport and storage proteins, sensors, and regulatory proteins [23,34]. The ability of carbon monoxide to affect cytochrome C even in very low concentrations could explain a possible regulatory role of CO in the switch from energy production from predominantly oxidative phosphorylation to glycolysis [35].

## 3. Carbon Monoxide and Mitochondria

Mitochondria are the major consumers of oxygen in cells, and normal function of this organelle is dependent on the electron transport chain. CO directly targets mitochondria by depriving this organelle of oxygen and binding with the end point of the ETC**—**cytochrome c [36,37]. This type of inhibition of the ETC of mitochondria leads to mitochondrial depolarisation in the same way as in chemical hypoxia, causing the production of ROS [9,38], and can be observed even in physiological CO-induced signalling [39]. CO-induced mitochondrial ROS production is dependent on the inhibition of respiration that stimulates electron leak in the ETC. Importantly, this effect could be blocked by the presence of the CO scavenger myoglobin [40], even in live tissues [41], although CO-induced mitochondrial ROS could play an anti-inflammatory role or be a switch between glycolysis and oxidative phosphorylation [26,39]. Mitochondrial ROS are mainly produced within the first minutes of CO exposure and induce oxidative stress, thus having an impact over the entirety of CO neurotoxicity [9]. It should be noted that the period of CO-induced mitochondrial ROS production is more prolonged and intensive than ROS production in mitochondria in response to chemical ischemia and oxygen/glucose deprivation [9,38]. Thus, toxic concentrations of CO inhibit cytochrome c oxidase, which leads to ROS production in the ETC of mitochondria. The results obtained from patients’ studies have shown alterations in mitochondrial respiration and increased reactive oxygen species production, i.e.**,** hydrogen peroxide, in patients poisoned with carbon monoxide [42]. Importantly, treatment of these patients with hyperbaric oxygen improved mitochondrial and cell respiration but dramatically increased the production of ROS, which confirms the role of mitochondrial ROS in CO toxicity and the mechanism of cell toxicity under hyperbaric oxygen in post-CO recovery [42]. It should be noted that people with mitochondrial mutations or who are affected by mitochondrial toxins might be more vulnerable to CO-induced toxicity. Thus, mitochondrial inhibitors should increase the production of hydroxyl radical in mitochondria during CO poisoning [43].

## 4. NADPH Oxidase and Carbon Monoxide Toxicity

NADPH oxidases encompass a family of enzymes which includes five types of NADPH oxidases (NOX 1–5) and two types of DUOX (1–2). In these brain cells—neurons, astrocytes, and microglia—the selective expression and function of NOX 2, 3, and 4 have been shown [44]. Activators of these enzymes initiate the assembly of the subunit proteins (membranal and cytosolic) into huge enzymatic complexes and trigger the production of superoxide or hydrogen peroxide with changes in the proton gradient along the plasma membrane. The activation of NADPH oxidase in CO poisoning has been shown for neurons and astrocytes [9,45]. CO activates this enzyme through P2Y receptors and cAMP or through calcium signaling in neurons and astrocytes that, in these cells, have shown to be triggered by glutamate or ATP release in the process of glutamate excitotoxicity [46,47,48]. Although NADPH oxidases produce superoxide or hydrogen peroxide, further transformation of ROS to the hydroxyl radical has been shown to be the trigger for CO toxicity [39].

However, NADPH oxidase can be assembled and activated even in anoxic conditions, but this enzyme cannot produce ROS if the oxygen level is below ~15 mmHg, the threshold required to enable it to bind to this enzyme. Considering this, in these scenarios, anoxia/re-oxygenation of CO/reperfusion, NADPH oxidase will start to produce ROS when the oxygen level is higher, i.e., during the time of re-oxygenation, making this period the most critical to neurons and astrocytes [19,38]. Interestingly, hyperbaric oxygen (HBOT), which is often used as an adjuvant therapy following immediate high flow oxygen therapy for severely CO-poisoned patients, maximises the immediate effects of CO toxicity, such as neuroinflammation, and NADPH oxidase activation [49,50].

## 5. Xanthine Oxidase and CO Toxicity

Hypoxia or CO-induced inhibition of mitochondrial respiration and oxidative phosphorylation leads to a reduction in ATP production in cells and stimulates the further activation and ultimately breakdown of purine nucleotides to xanthine. It induces the activation of ROS production in xanthine oxidase [51]. Interestingly, this enzyme is able to produce superoxide even in complete anoxia [38]. Thus, the presence of carbon monoxide induces an even more pronounced inhibition to mitochondrial bioenergetics than hypoxia, and the activation of xanthine oxidase and ROS production is also shown to occur earlier [9,38]. The role of xanthine oxidase in the mechanism of CO-induced ROS production was shown over 40 years ago [52,53]. Importantly, the inhibitor of XO, allopurinol, has been shown to reduce the severity of delayed neurologic sequelae in an experimental carbon monoxide toxicity model [54], confirming the importance of this enzyme in the mechanistic development of pathology due to CO neurotoxicity.

## 6. Monoaminoxidase and Carbon Monoxide

The enzymes monoaminoxidase A and B utilise monoamines and control the level of neurotransmitters and neuromodulators, such as dopamine, noradrenaline, adrenaline, and serotonin [55]. MAOs produce aldehydes and hydrogen peroxide. Interestingly, CO-induced production of ROS at the time of re-oxygenation with hyperbaric or atmospheric oxygen could be blocked by the monoamine oxidase (MAO) inhibitor pargyline [56]. MAO B inhibitors have also been shown to be protective against CO poisoning [57]. The production of ROS in MAO in conjunction with CO poisoning could be explained by a higher release of monoamines at the time of carbon monoxide exposure [57]. It should be noted that monoamine oxidation, including dopamine oxidation, could be an additional inductor for cell death in CO-related neurotoxicity [58,59].

## 7. CO Toxicity by Lipid Peroxidation-Driven Ferroptosis

Acute CO poisoning leads to a massive overproduction of ROS via different enzymatic and non-enzymatic mechanisms, leading to lipid peroxidation of plasma and intracellular membranes as an end point of oxidative damage [9,60]. Ultimately, increased rates of lipid peroxidation lead to the collapse of cellular membranes and thus collapse the function of intracellular organelles and the cell as a whole [61]. Interestingly, an increase in the physiological way of CO production by heme oxygenase in elderly people might be responsible for free radical production and lipid peroxidation and is suggested to be one of the possible explanations of the process of ageing [62].

Lipid peroxidation has been known for its implication in a type of cell death called ferroptosis [63]. CO toxicity has been shown to include a lipid peroxidation phase [52,64,65]. Inhibitors of lipid peroxidation could alleviate cell death in a rat model of CO poisoning [66]. Moreover, inhibitors of ferroptosis have been shown to be neuroprotective in this setting [67].

## 8. CO Poisoning Oxidative Stress and Cellular Antioxidant Parameters

During CO poisoning, and especially in the re-oxygenation phase, the rate of free radical production and lipid peroxidation, respectively, increases [9].

Higher levels of urine 8-hydroxy deoxyguanosine (8-OHdG), plasma protein carbonyl (PK), and malondialdehyde (MDA) have been detected as a readout for the severity of oxidative stress in patients with CO poisoning [68] (see Figure 1).

Decreased superoxide dismutase (SOD) and catalase (CAT), and increased glutathione peroxidase (GPx) activities and lower GSH levels or release of GSH from erythrocytes, have been reported for patients with CO poisoning and for cell models [9,68,69].

## 9. Sequence of Activation of ROS Production from Various Enzymatic Sources in the Mechanism of CO-Induced Neurotoxicity

The role of CO-induced oxidative stress in the mechanism of neuronal loss has been discussed for several years with a growing number of enzymes involved in this process described in the literature. However, a therapy with antioxidants, as with a large number of other pathologies, is effective only on the level of isolated cells; with some positive but not completely protective effects in vivo [70,71]. There is no doubt about the involvement of reactive oxygen species production and oxidative stress in the mechanism of CO-induced neuronal cell death. We suggest that this could partially be connected to a sequence of events which we have found and have described recently [9]. Thus, the activation of the mitochondrial ROS production appears just for a short time (~5–10 min) as CO exposure commences and when the electron transport chain of mitochondria is not yet completely inhibited. This could explain the relatively low impact of mitochondrial antioxidants in the neuronal protection against CO toxicity. Xanthine oxidase is activated and produces ROS only after a profound energy depletion (25–30 min of CO exposure) with a sufficient impact on neuronal toxicity that could be confirmed by a number of studies with effective cell protection using an inhibitor of XO—allopurinol [9,54]. Mitochondrial or general antioxidants or inhibitors of XO oxidase are very promising agents against CO toxicity but have one serious limitation [71,72]: all these compounds could exhibit its protective effects only during CO exposure. For this reason, they could be used only as a pretreatment. Although the assembling and activation of NADPH oxidase is also occurs during CO exposure, massive ROS production starts only at the time of re-oxygenation [9,19,38]. Potentially, it might help to use NADPH inhibitors before re-oxygenation or the use of HBOT. However, the potential for using this strategy is limited by the lack of effective NADPH inhibitors which could be used in the clinical setting.

## 10. Strategies to Overcome CO-Induced DNS

Patients with CO poisoning are treated using oxygen therapy to restore oxygen partial pressure in tissues, especially in the brain and heart. It is the most immediate treatment and instantaneously starts the mechanisms for recovery from CO poisoning; it is thus obligatory and inevitable. However, considering that the most critical stage with highest ROS production occurs during the reperfusion phase [9], it is necessary to address the massive and highly damaging phase of ROS production through different supplements during reperfusion to avoid neuronal loss and subsequent DNS often associated with CO poisoning. In cell models, several approaches have been proven to be effective in reducing oxidative damage and increase the survival of brain cells. Mostly, inhibitors of ROS-producing enzymes such as allopurinol [54,73] and antioxidants such as NAC, methylene blue, resveratrol, amantadine, and curcumin [74,75,76,77,78,79] are shown to be powerful tools to correct for oxidative damage during the reperfusion stage. Developments in methods for CO removal**,** such as extracorporeal membrane oxygenation, are very promising, but taking into account the results of studies pointing towards increased oxidative stress in the reperfusion phase, this method could be improved with the simultaneous addition of inhibitors of the various sources of oxidative damage [80].

Although intravenous GSH has been shown to be protective against reperfusion injury [81], this method should undergo future development due to the impermeability of GSH through biological membranes or the blood–brain barrier (BBB) [82]. However, the development of cell-permeable GSH derivatives and future improvements in detection methods for GSH assessment may further help to utilise this therapeutic strategy against CO-induced oxidative damage [83].

It is known that the upregulation of Nrf-2 gene is induced and controls cell survival during CO poisoning [84] (see Figure 1). Thus, the correction of oxidative damage by Nrf-2 activators’ application (N-butylnaphtide, sulforaphane, edaravone) at the time of reperfusion has been shown to be neuroprotective in rat models of CO poisoning [85,86,87]. One of the possible problems of utilising Nrf-2 activators against CO-induced oxidative damage and neuronal loss is that this type of activator should be introduced prior to exposure to CO to enable the expression of downstream effectors. Introduction at the time of tissue re-oxygenation might be unsuccessful due to insufficient time for activity to be effective.

Combined effects of certain compounds working both as antioxidants and nitric oxide synthase (NOS) inhibitors, such as Ginaton or methylene blue, have been proven to be effective in acute carbon monoxide poisoning [76,88,89].

Corticosteroids have been used to reduce CO-induced neuroinflammation, either as a monotherapy or combined with other therapies. In particular, dexamethasone [90], methylprednisolone [91,92]**,** and dehydroepiandrosterone/pregnenolone [93] have been proven to be effective in the reduction in the severity of delayed neurological sequelae.

## 11. Conclusions

Carbon monoxide poisoning is a global cause of unintentional morbidity and mortality. It is associated with a reduction in oxygen delivery to the tissues. Clearly, the provision of a high flow of 100% oxygen is the standard treatment for CO poisoning. However, evolving scientific evidence is highlighting that the known neurological damage associated with hypoxic tissue injury and subsequent treatment with supplementary oxygen in pre-hospital and hospital environments is, in cases of CO exposure, showing far greater damage to brain cells in vitro and in vivo than expected. CO is involved in cellular free radical metabolism and exposure to exogenous sources of CO are able to disrupt the mechanisms that endogenously produced CO seeks to protect and maintain.

## Abbreviations

ETC	electron transport chain
MAO	monoamine oxidase
ROS	reactive oxygen species
OXPHOS	oxidative phosphorylation
HO-1	haem oxygenase 1
COHb	carboxyhaemoglobin
CYP	cytochrome P450-dependent monooxygenases
Nrf-2	nuclear factor erythroid 2-related factor 2
NOX	NADPH oxidase
DUOX	dual oxidase
P2Y	purinoreceptor Y type
HBOT	hyperbaric oxygen therapy
NAC	nicotinamide cysteine
DNS	delayed neurological sequelae
XO	xanthine oxidase
